# Approaches to Improve the Surveillance, Monitoring, and Management of Noncommunicable Diseases in HIV-Infected Persons: Viewpoint

**DOI:** 10.2196/10989

**Published:** 2018-12-20

**Authors:** Pragna Patel, Keith Sabin, Peter Godfrey-Faussett

**Affiliations:** 1 Centres for Disease Control and Prevention Atlanta, GA United States; 2 Joint United Nations Programme on AIDS Geneva Switzerland; 3 Department of Clinical Research London School of Hygiene and Tropical Medicine London United Kingdom

**Keywords:** HIV, noncommunicable diseases, systems integration, epidemiologic surveillance

## Abstract

Low-income and middle-income countries (LMICs) are undergoing an epidemiological transition, in which the burden of noncommunicable diseases (NCDs) is rising and mortality will shift from infectious diseases to NCDs. Specifically, cardiovascular disease, diabetes, renal diseases, chronic respiratory diseases, and cancer are becoming more prevalent. In some regions, particularly sub-Saharan Africa, the dual HIV and NCD epidemics will pose challenges because their joint burden will have adverse effects on the quality of life and will likely increase global inequities. Given the austere clinical infrastructure in many LMICs, innovative models of care delivery are needed to provide comprehensive care in resource-limited settings. Improved data collection and surveillance of NCDs among HIV-infected persons in LMICs are necessary to inform integrated NCD-HIV prevention, care, and treatment models that are effective across a range of geographic settings. These efforts will preserve the considerable investments that have been made to prevent the number of lives lost to HIV, promote healthy aging of persons living with HIV, and contribute to meeting United Nations Sustainable Development Goals.

## The HIV and NCD Syndemic

In high-income countries, antiretroviral therapy (ART) has improved the survival of persons living with HIV (PLHIV), resulting in declines in morbidity and mortality and a shift in the natural history of HIV disease [1-3]. With ART scale-up in the low-income and middle-income countries (LMICs), similar epidemiologic transitions are expected, resulting in an expanding and aging HIV population [4,5]. Since 2004, the US President’s Emergency Plan for AIDS Relief (PEPFAR), in collaboration with local governments, has established HIV prevention, care, and treatment programs in over 30 countries worldwide [6], and the Global Fund to Fight HIV, Tuberculosis and Malaria has funded programs in more than 100 countries, including some PEPFAR-funded countries [7]. Globally, the provision of ART accelerated dramatically over the past decade, supporting 19 million PLHIV on ART through June 2016. When the Joint United Nations Programme on AIDS (UNAIDS) [8] 90-90-90 goals (90% of people with HIV diagnosed, 90% of them on ART, and 90% of them virally suppressed by 2020) are realized, AIDS-related opportunistic infections will become rare [9], and noncommunicable diseases (NCDs) will become increasingly prevalent among PLHIV [10-16].

The natural history of HIV for persons who are stable on ART is that HIV becomes a chronic disease with an increased risk of chronic comorbidities. These chronic conditions include, but are not limited to, cardiovascular disease [17], depression [18-20], cancers [21,22], and metabolic abnormalities, including insulin resistance with consequent dyslipidemia, type 2 diabetes, and lipodystrophy [23-25]. The increased prevalence of NCDs among HIV-infected adults reflects a combination of factors, including aging, a greater prevalence of traditional risk factors, infection with oncogenic viruses, direct consequences of HIV (eg, inflammation), and exposure to specific antiretrovirals [26-36]. A recent systematic review examined the prevalences of the following 4 NCDs (and their risk factors) in LMICs that are known to commonly occur among PLHIV: cardiovascular disease, cervical cancer, mental health, and type 2 diabetes [37]. However, data were sparse for some diseases entities.

Increasing patient awareness of the effects of long-term HIV infection and effective levels of antiretroviral treatment, that is, viral suppression, particularly the shift to a predominance of chronic diseases, is important because this improved awareness may prompt patients to report symptoms and request screening for related conditions. Therefore, patient education can improve our ability to detect NCDs as they become more prevalent. An effective public health response to HIV, now that the epidemic is maturing, requires treatment and prevention of NCDs among PLHIV in LMICs. It is imperative to understand the predominant risk factors, the consequent symptoms and complications, and the available appropriate treatments and preventative interventions for each NCD.

If left unaddressed, NCDs may undermine the gains in healthy life-years realized by global health investments in HIV prevention and treatment [38]. The emerging NCD epidemic presents a unique opportunity to leverage the tremendous investments made in the existing HIV health platforms so that these enhanced health systems can deliver improved HIV care, which includes the prevention and treatment of chronic comorbidities to achieve further reductions in preventable deaths. Additionally, lessons learned from the public health approach in response to the HIV epidemic could be applied to the care and treatment of NCDs, given the chronic nature of both conditions. To understand this emerging syndemic, improved surveillance and clinical monitoring of NCDs among PLHIV are necessary to estimate the burden of and risk factors for NCDs among HIV-infected persons.

HIV surveillance has been shifting with the epidemic response over the past 2 decades. Reliance on sentinel surveys and AIDS case reporting was augmented with household surveys, community-based surveys, and HIV case reporting. The current trend is to move toward more routine data collection activities. Patient monitoring systems that capture a variety of health conditions, such as routine perinatal mother-to-child transmission surveillance, are becoming more important as increasing numbers of PLHIV are accessing care and treatment. Some comorbidities are already recognized as important to monitor, though these are primarily infectious, such as tuberculosis, hepatitis B, and hepatitis C. As effective ART continues to be scaled up across LMICs, chronic conditions will become more important to monitor because more PLHIV will achieve virologic suppression.

National HIV program responses would benefit from the integration of screening for NCDs known to be associated with or closely linked to HIV, for example, human papillomavirus-related cancers, such as cervical cancer, and cardiovascular disease [37]. As indicated by a recent systematic review, current efforts are limited to clinical cohorts or studies [37]. Routine screening is not presently being conducted in many clinical settings in sub-Saharan Africa (SSA) that offer HIV care; however, some efforts do exist [39]. General screening could include blood pressure measurement, height and weight assessment, lifestyle counseling including smoking cessation and physical activity promotion, tests for liver and kidney function, and cervical cancer screening. Given the similarities between HIV and NCD care and management, a coordinated approach to address both seems feasible and warranted [40]. Additionally, the resultant health systems’ strengthening will facilitate improvements in health care coverage worldwide.

## Benefits of Noncommunicable Diseases-HIV Integration

The PEPFAR HIV program has been criticized as a vertical disease-specific health system with regards to funding, supply chain, and clinician staffing [41-43]. Horizontal integration avoids these issues by taking a multi-disease approach to care [43,44]. Models such as chronic disease clinics have proven effective in treating diabetes, hypertension, and HIV and allow caregivers to take a patient-centered approach in responding to the health needs of PLHIV [45]. By preventing late-stage NCD presentation, horizontally-integrated care models minimize NCD-related mortality and may thus maximize cost- effectiveness if cost-prudent integration is employed [38]. Integrated models of care have also shown increased retention of patients with comorbid disease, thereby conferring improvements in adherence to treatment and continuity of care [41,43]. Lastly, integration of HIV and NCD monitoring and evaluation (M&E) systems allows for improved data collection and analysis pertaining to NCDs, which could then be used to support large-scale health policy change [46].

## Barriers to Integration

The integration of NCD and HIV health care can lead to improvements in the quality of care and treatment; however, this enhancement of service provision can be costly. Initial overhead costs needed for service integration include training of clinical staff, procurement and distribution of laboratory reagents and medications, and greater supply chain needs. These costs can often be daunting for policy makers in resource-limited settings [47]. In integrated care systems, deficits in human resources for health, such as limited numbers of health care workers, can become problematic owing to the need for increased requirements for clinical services. Deficits here can lead to bottlenecking because of high patient loads and increased responsibilities for few clinicians [48,49]. Task-shifting can alleviate this bottleneck, but the cost of additional staff and training may be prohibitive.

Economically, integrating NCD care into LMIC HIV systems is only viable with buy-in from the government, external donors, and national constituents. As with PEPFAR for HIV care, countries may require infrastructural capacity building before they become capable of supporting these larger health care systems on their own [50]. Although not universally feasible, an ideal solution would entail HIV programs providing the necessary infrastructure for NCD programs with multi-sectoral support and coordination, thus minimizing cost [51]. As countries transition from low-income to middle-income, an additional challenge is finding resources to support HIV programs as donor resources diminish in the face of competing health care demands. These demands include the need for additional financial and human resources in constrained situations. Finally, the integration of separate health systems creates a need for improved management practices. Standards to address selection biases, validity, and reliability of data sources are needed to ensure that robust data are collected. This will ensure that data from a variety of sources (eg, clinical systems, research, and M&E) are standardized to some extent and will limit missing and unreliable data. M&E systems must be expanded to include NCD screening and treatment services, and quality of care must be ensured through continued monitoring, evaluation, and medical education [43,49,52].

As NCDs become more prevalent, resource allocation needs to be informed by knowledge of the burden of disease and evidence-based interventions. Cost-effective models of comprehensive care of HIV, a chronic disease when viral suppression is achieved and maintained, that integrates management of NCDs associated with HIV are needed. The first step is leveraging surveillance systems to enhance our knowledge of the NCD burden among PLHIV by collecting information on risk factors and related morbidity. Several strategies to improve knowledge of disease burden exist, allowing public health officials to design or enhance interventions to reduce years of life lost. Among these are case-based surveillance and population-level monitoring systems; registries, which use medical records data for clinical surveillance; cohort monitoring in LMICs; and population-based surveys.

## Case-Based Reporting and Population-Level Patient Monitoring Systems

HIV case reporting has been a part of second generation surveillance, as proposed by the World Health Organization (WHO) and UNAIDS, since 2000 [53]. Case-based reporting systems remain unevenly developed across LMICs [54,55]. The rapid expansion of ART and the promulgation of the 90-90-90 treatment targets have renewed the focus on case reporting, linked to patient monitoring systems, by WHO and UNAIDS. In settings where unique identifiers are available, HIV case report systems can be crossed with registries where they exist to link associated conditions; currently, cancer registries are the most prevalent [56]. Every effort should be made to protect patient privacy and ensure confidentiality as individual systems grow to collect more data elements and are linked to other systems. To strengthen overall patient care, the population-level HIV patient monitoring system, which is designed to track ART adherence and HIV viral suppression, can monitor conditions associated with both HIV infection and long-term exposure to ART. Population-level patient monitoring systems can capture risk factors such as hypertension and hyperlipidemia as well as outcomes such as cardiovascular disease and cancer [57]. Specific conditions that have a demonstrated link to HIV infection or long-term use of ART should be collected and reported, and appropriate responses should be developed for the patient and the population.

## Registries

Registries collect clinical information of all patients diagnosed with a certain condition within a particular catchment area over a period of time and are particularly useful for patient care, especially in areas that lack population-level patient monitoring systems. Patients’ information from participating clinics is entered into the registry after diagnosis of a particular NCD or identification of a person with previously diagnosed NCD(s) of interest; this registry contains all patients under the same participating health care provider or in the same facility who have the same NCD. The clinic data collected at each visit is entered into the registry or, ideally, automatically flows from the patients’ medical records into the registry without additional resources required for data entry[58]. These data can then be aggregated at the regional or national level and can be used for cohort monitoring; program evaluation; tracking indicators for accountability to funders, policy makers, and stakeholders; and for performance reporting. Furthermore, at the patient level, registries can be used to track adherence, provide reminders for follow-up and preventative services as well as track health changes over time [59].

Registries range from a low-tech paper-based format to a higher-tech electronic format. An electronic medical records (EMRs) system is ideal because it allows for the data to be collected in real-time from a linked EMRs system, and individual or cohort outcomes can be more easily monitored. However, an EMR is not necessary [60]. As the number of cases grows, as expected for an NCD of interest, paper-based registries cannot work efficiently or accurately. Collecting patient data and manually creating a registry is time-consuming and takes health care staff away from patient care [61]. However, it is still a useful modality, and the lack of EMRs systems should not preclude the development of registries, at least in part, for improving patient care.

The creation of registries is important and prudent because the current management of NCDs is, in many places, unstructured and unmonitored. Through the creation of registries, health care systems can gain the ability to collect data on NCDs [62]. Reliable patient data at all levels are necessary for surveillance, forecasting drug and commodity procurement, human resource needs, and logistics required to keep the program on track [63]. However, it is important to ensure that any new data collection requirements render useful information and do not create an additional burden for the health care staff. Registries can also be structured to give feedback on performance, which serves as a benchmark to determine the quality of care and quality improvement at the level of individual providers, health care teams, or clinics. Registries can also be used to support clinical decision making; to allow providers to be proactive rather than reactive, for example, by setting up patient reminders about needed services or follow-up visits; and to share data throughout a practice, among practices, and potentially throughout an entire health care system.

Ethical considerations may prevent the inclusion of name-based HIV status in a registry, requiring unique identifiers. However, even with unique identifiers, confidentiality may be lost if systems are not created with checks to ensure privacy. Therefore, precautions to protect the identity of individuals in both name-based HIV case report databases as well as databases that use unique identifiers must be taken, particularly in contexts where stigma and discrimination against PLHIV are strong.

## Cohort Monitoring

An important use of registries is cohort monitoring [64], which is inspired by directly observed treatment, short-course for tuberculosis and provides a useful way of assessing whether interventions, as specified in the country guidelines for NCDs, are being performed as well as to track performance and progress of the intervention. This allows the country to review the progressive scale-up of those alive and on treatment and see where these patients are being treated (hospital, urban health center, rural health center, or private clinic). Furthermore, individual patient data can be deidentified and aggregated to determine morbidity and mortality levels for NCDs generally, generate incidence and prevalence rates of complications, and assess performance at local and national levels [59]. Successful application of the directly observed treatment, short-course monitoring system to patients with diabetes in Malawi [58] and those with hypertension in Jordan [61] has been reported.

## Population-Based Surveys

The WHO-UNAIDS Technical Working Group on HIV Surveillance has advocated the use of population-based surveys to understand the risk factors for HIV and to determine HIV incidence and prevalence. As the HIV epidemic matures in SSA, these surveys should include questions about NCDs and related risk factors. Basic screening for diseases such as hypertension would be easy to employ as well [65]. Household surveys such as the Demographic and Health Surveys (DHS) collect data on screening for NCDs and HIV. Some DHS include testing for NCDs and HIV [66,67]; however, it is uncommon for DHS to routinely report on any associations. These data are publicly available and can be examined for associations, but this should become part of routine public health reporting. Additionally, the WHO STEPwise approach to surveillance should consider an HIV module for high-burden countries. This would allow for both general population and HIV-specific estimates of NCD burden, which would inform national policies and plans to address the growing NCD burden.

The PEPFAR-funded Population-based HIV Impact Assessment surveys are nationally representative, household-based HIV surveys that are used to provide subnational estimates for HIV prevalence and viral load suppression and national estimates for HIV incidence to measure the status of the HIV epidemic and impact of HIV prevention and treatment programs [68]. Data on demographic characteristics, risk behaviors, and testing and treatment history are collected through household and individual questionnaires. These surveys can also be leveraged to collect data about NCDs by including questions about NCD risk factors and diagnosed NCDs. Additionally, HIV testing is routinely performed with laboratory capacity to test blood samples, which provides an opportunity for screening for NCDs as well.

## Mathematical Modeling

Given the limited data currently available concerning the NCD burden among PLHIV in LMICs, mathematical modeling could provide useful information about prevalence. The granularity of the prevalence rates may be limited based on available country-specific data for the models. However, with concerted efforts to collect meaningful data to inform these models, relevant figures could be generated. Currently, the Global Burden of Disease Study generates estimates for the prevalence of various diseases and has a Web-based tool that generates visualizations of these data [69]. As a next step, it may be imperative to develop a tool to generate the estimates of coburden of certain diseases such as HIV and tuberculosis or HIV and cardiovascular disease. Data of coburden can help us to identify syndemics and thus better focus our efforts and resources to prevent and treat these diseases.

## Conclusions

With the success of the global effort to scale up ART access, LMICs, particularly those in SSA, in which mortality has been dominated by HIV over the past decades, will start to experience syndemics. Increasingly, mortality attributable to NCDs will be greater than communicable diseases as it is in the rest of the world. Improved data collection and surveillance of NCDs among HIV-infected persons in LMICs are necessary to inform integrated NCD-HIV prevention, care, and treatment models that are effective across a range of geographic settings. Implementation of integrated care will strengthen current health systems and facilitate a platform for more comprehensive and less fragmented health care delivery as well as M&E systems. These efforts will preserve the considerable investments that have been made to prevent lives lost to HIV, promote healthy aging of PLHIV, and contribute to meeting United Nations Sustainable Development Goals [70]. Additional incremental investments in NCD management among PLHIV could broaden health care coverage and support a research agenda, which would benefit both PLHIV and the general population. Furthermore, as countries start to achieve HIV epidemic control, the integration of HIV and NCD management will provide a transition plan for extending the comprehensive care provided to PLHIV to the general population; this will facilitate the goal of improved overall access to health care.

## Abbreviations

ART: antiretroviral therapy
DHS: Demographic and Health Surveys
EMR: electronic medical record
LMIC: low-income and middle-income countries
M&E: monitoring and evaluation
NCD: noncommunicable diseases
PEPFAR: President’s Emergency Plan for AIDS Relief
PLHIV: persons living with HIV
SSA: sub-Saharan Africa
UNAIDS: Joint United Nations Programme on AIDS
WHO: World Health Organization
